# A novel nomogram for predicting prolonged mechanical ventilation after acute type A aortic dissection surgery: a retrospective study investigating the impact of ventilation duration on postoperative outcomes

**DOI:** 10.1080/07853890.2024.2392871

**Published:** 2024-08-22

**Authors:** Luo Yuanxi, Zeshi Li, Xinyi Jiang, Yi Jiang, Dongjin Wang, Yunxing Xue

**Affiliations:** a Department of Cardiovascular Surgery, Nanjing Drum Tower Hospital, Chinese Academy of Medical Sciences & Peking Union Medical College, Peking Union Medical College Graduate School, Nanjing, China; b Peking Union Medical College Hospital, Chinese Academy of Medical Sciences & Peking Union Medical College, Beijing, China; c Department of Cardiovascular Surgery, Nanjing Drum Tower Hospital, Affiliated Hospital of Medical School, Nanjing University, Nanjing, China

**Keywords:** Aortic dissection, mechanical ventilation, mortality, enhanced recovery

## Abstract

**Objective:**

Acute type A aortic dissection (ATAAD) is a devastating cardiovascular disease with extraordinary morbidity and mortality. Prolonged mechanical ventilation (PMV) is a common complication following ATAAD surgery, leading to adverse outcomes. This study aimed to investigate the correlation between mechanical ventilation time (MVT) and prognosis and to devise a nomogram for predicting PMV after ATAAD surgery.

**Methods:**

This retrospective study enrolled 1049 ATAAD patients from 2011 to 2019. Subgroups were divided into < 12 h, 12 h to < 24 h, 24 h to < 48 h, 48 h to < 72 h, and ≥ 72 h according to MVT. Clinical characteristics and outcomes were compared among the groups. Using multivariable logistic regression analyses, we investigated the relationship between each stratification of MVT and mortality. A nomogram was constructed based on the refined multivariable logistic regression model for predicting PMV.

**Results:**

The total mortality was 11.8% (124/1049). The results showed that the groups with MVT 48 h to < 72 h and ≥ 72 h had significantly higher operative mortality compared to other MVT categories. Multivariate logistic regression analysis showed that MVT ≥72 h was significantly associated with higher short-term mortality. Thus, a nomogram was presented to elucidate the association between PMV (MVT ≥72 h) and risk factors including advanced age, preoperative cerebral ischemia, ascending aorta replacement, concomitant coronary artery bypass grafting (CABG), longer cardiopulmonary bypass (CPB), and large-volume intraoperative fresh frozen plasma (FFP) transfusion. The nomogram exhibited strong predictive performance upon validation.

**Conclusions:**

Safely extubating patients within 72 h after ATAAD surgery is crucial for achieving favorable outcomes. The developed and validated nomogram provides a valuable tool for predicting PMV and optimizing postoperative care to improve patient prognosis. This novel nomogram has the potential to guide clinical decision-making and resource allocation in the management of ATAAD patients.

## KEY MESSAGES

Prolonged mechanical ventilation (PMV) is a common complication following ATAAD surgery, leading to adverse outcomes.

Safely extubating patients within 72 hours after ATAAD surgery is crucial for achieving favourable outcomes.

A novel, validated nomogram incorporating risk factors such as age, comorbidities and intraoperative factors predicts PMV after ATAAD surgery, aiding clinical decision-making and optimizing postoperative care.

## Introduction

Acute type A aortic dissection (ATAAD) is a severe cardiovascular disease associated with high morbidity and mortality rates. Prompt surgical intervention is imperative for patients with ATAAD to avoid devastating outcomes [1,2]. Surgery is indispensable and offers a critical lifeline for these patients. Mechanical ventilation plays a crucial role in assisting patients through the turbulent initial stage following surgery [3]. Despite advancements in surgical techniques and perioperative strategies, prolonged postoperative mechanical ventilation remains prevalent among patients undergoing ATAAD repair, leading to significant utilization of medical resources [2,4]. Moreover, patients who experienced short-term mortality following ATAAD surgery typically had extended mechanical ventilation time (MVT), as well as higher reintubation and tracheotomy rates, compared to survivors [5,6]. These observations highlight the need for further exploration of the relationship between MVT and mortality, as well as the identification of high-risk patients’ clinical characteristics. This knowledge can facilitate the timely implementation of appropriate interventions to achieve favourable prognoses.

MVT can serve as a surrogate marker for assessing the critical condition of patients after ATAAD surgery and is considered an important quality indicator by the Society of Thoracic Surgeons (STS) [7]. Various factors contribute to the prolonged need for mechanical ventilation in these patients. For instance, postoperative complications such as pulmonary insufficiency, neurological issues, and the need for re-exploration often hinder early extubation [3,8,9]. From one perspective, the advent of enhanced recovery programmes (ERPs) for cardiac surgery emphasizes the importance of early weaning from mechanical ventilation to facilitate postoperative mobility and feeding [10]. The medical staff should actively support patients in achieving early extubation as part of these programmes. From another perspective, several characteristics of ATAAD contribute to the extended duration of mechanical ventilation. Firstly, the systemic inflammatory response triggered by ATAAD activates pulmonary insufficiency [11]. Additionally, the use of prolonged cardiopulmonary bypass (CPB) and deep hypothermia circulatory arrest (DHCA) during ATAAD surgery leads to pulmonary hypoxia and ischemia [4,12]. Lastly, surgical trauma can exacerbate lung inflammation.

Numerous studies have investigated the duration of mechanical ventilation following cardiac surgery [13,14]; however, only a limited number have specifically included patients with ATAAD [4]. Furthermore, there exists divergence in the definition of prolonged mechanical ventilation (PMV) in patients who have undergone ATAAD repair [5,9]. Our objective is to elucidate the correlation between MVT after ATAAD surgery and operative mortality, as well as identify high-risk factors associated with an extended need for ventilation.

## Materials and methods

### Study cohort and definition

This study was a single-centre, retrospective study that included a cohort of 1049 adults who underwent surgery for acute type A aortic dissection. The study protocol was approved by the Ethics Committee of Nanjing Drum Tower Hospital (reference number: 2020-185-01). Given the retrospective nature of the study, the requirement for individual consent was waived. The collection of clinical information from the patient population adhered strictly to the guidelines set forth in the Declaration of Helsinki (seventh revision, 2013). The work adheres to the strengthening the reporting of cohort, cross-sectional and case–control studies in surgery (STROCSS) criteria [15].

The primary endpoint of our study was operative mortality, which was defined as any death that occurred during hospitalization, regardless of the time frame, or deaths within 30 days post-discharge. The secondary endpoints included MVT, PMV (MVT ≥72 h), postoperative tracheotomy, haemorrhagic stroke, ischaemic stroke, paraplegia, GI bleeding, limb ischaemia, bowel ischaemia, surgical site infection and re-exploration. Postoperative MVT was defined as the duration from the completion of surgery to the first extubation, excluding any subsequent reintubation periods. To classify the duration of mechanical ventilation, we adopted predefined subgroups based on ­previous literature and the clinical experience of our centre [3,5,9]. These subgroups were defined as ­follows: < 12 h, 12 h to < 24 h, 24 h to < 48 h, 48 h to < 72 h, and ≥ 72 h.

*Inclusion criteria:* (1). Adults (≥18 years old) with imaging-confirmed AAD; (2). Underwent aortic surgery requiring CPB. *Exclusion criteria:* (1). Intraoperative death; (2). Pregnancy; (3). Severe comorbidities (e.g. lung cancer); (4). Missing key outcome data (short-term mortality, mechanical ventilation duration, reintubation, etc.).

### Surgical approach

For patients with a confirmed diagnosis of ATAAD, emergency surgery will be performed for all cases, except for those with associated organ malperfusion, who undergo pre-emptive reperfusion treatment. The surgical approach has been extensively detailed in our previous articles [16,17]. In essence, we determine the method of cannulation, extent of aortic replacement and cerebral perfusion based on the involvement of dissection. The choice of additional procedures, such as coronary artery bypass grafting (CABG), valve replacement, etc. depends on whether the coronary arteries are suffered or if valve diseases are present. Following the establishment of CPB, aortic crossclamp is initiated and myocardial protection involves both antegrade and retrograde perfusion. DHCA is performed when the core temperature and nasopharyngeal temperature drop to 22–24 °C. Depending on the cannulation placement, either antegrade cerebral perfusion, retrograde cerebral perfusion, or no cerebral perfusion will be selected. Different surgical approaches for the root and arch are chosen based on the involvement of these areas. After completion of the surgical procedure, aortic clamping is discontinued, followed by haemostasis and chest closure.

### Respiratory weaning protocol

Following surgical procedures, all patients returning to the ICU undergo comprehensive assessments by healthcare providers. These assessments encompass evaluations of drainage, systemic haemodynamics and neurological status, pivotal in determining the most suitable ventilation assistance strategy. Should a patient present alert, devoid of signs indicating organ malperfusion or bleeding risks, they progress into the transitional ventilation phase. Upon the restoration of spontaneous breathing, the ventilation mode transitions to continuous positive airway pressure (CPAP). Subsequently, a period on CPAP ensues, during which the patient’s stability is monitored, considering factors such as haemodynamics, the absence of excessive bleeding, normothermia, full consciousness, complete muscle recovery, normal acid–base balance, tidal volume surpassing 6 mL/kg, respiratory rate within the range of 10 to 30 breaths per minute, and arterial carbon dioxide pressure below 50 mmHg. Contemplation of extubation arises if these criteria are met [4,18]. However, in instances of post-extubation hypoxemia, various interventions may be implemented to enhance arterial oxygen pressure (PaO_2_). These interventions may include non-invasive positive pressure ventilation, high-flow oxygen therapy, inhalation of nitric oxide, or prone ventilation [19].

Tracheostomy is contemplated under several circumstances: PMV, instances where upper airway obstruction is bypassed, predicted difficulties with reintubation, repeated intubation, one or more unsuccessful extubation attempts, and the necessity to clear lung secretions along the tracheal pathway [6].

### Data analysis

Statistical analyses for this study were conducted using SPSS version 27.0 (IBM, USA) and R version 4.2.1. A significance level of less than 0.05 was considered statistically significant for all two-sided *P*-values. Depending on the normal distribution of continuous variables, either Student’s *t*-test or Mann–Whitney U-test was performed, with results presented as means with standard deviations or medians with interquartile ranges. Categorical variables were expressed as frequencies and percentages. Between-group differences in categorical variables were assessed using the chi-squared test or Fisher’s exact test. To evaluate the predictive values of continuous variables and establish dichotomization thresholds, ROC curve analyses were carried out.

Violin plots were employed to visualize the differences in continuous MVT between survivors and non-survivors. Furthermore, MVT was divided into five subgroups, and intergroup comparisons were conducted to analyse the variation in mortality between each group and the group with MVT < 12 h. Potential variables related to the preoperative, intraoperative, and postoperative phases were included in a backward stepwise multivariable logistic regression analysis to identify risk factors associated with operative mortality. Risk factors for prolonged ventilation were further explored using multivariable logistic regression, and the findings were incorporated into a nomogram model. The concordance index (C-index), calibration curve, and decision curve analysis (DCA) were employed to assess the performance and applicability of the developed model.

## Results

### Characteristics of the study population

A cohort of 1049 patients who underwent surgery for ATAAD was analysed, among whom 124 patients (11.8%) deceased. The comprehensive analysis delineated the characteristics of the entire population and discrepancies between survivors and non-survivors (Table 1). Preoperative, intraoperative, and postoperative data underscored that non-survivors exhibited a higher mean age (57.43 ± 13.78 vs 52.42 ± 13.02, *p* < 0.001) and presented with a greater prevalence of preoperative malperfusion syndromes, such as cardiac tamponade [27 (21.77%) vs 93 (10.05%), *p* < 0.001] and limb ischaemia [25 (20.16%) vs 125 (13.51%), *p* = 0.047]. Moreover, a larger proportion of non-survivors demonstrated preoperative myocardial ischaemia and underwent concurrent CABG during ATAAD repair [40 (32.26%) vs 163 (17.62%), *p* < 0.001; 21 (16.94%) vs 47 (5.08%), *p* < 0.001]. Receiver operating characteristic (ROC) curve analyses were conducted to assess the predictive values of intraoperative transfusions, including red blood cells, platelets, fresh frozen plasma, cryoprecipitate, as well as CPB time, aortic clamp time, and DHCA time concerning operative mortality (refer to Figure 2). The optimal cut-off values were employed for binary classification of the respective variables. Analysis demonstrated that deceased patients were prone to prolonged CPB and aortic cross-clamp times [241.00 (208.00 − 293.00) vs 222.50 (187.75 − 264.00), *p* < 0.001; 163.00 (137.75 − 216.50) vs 156.00 (125.00 − 193.00), *p* = 0.300]. Furthermore, non-survivors received higher volumes of blood transfusions during surgery. Additionally, a higher likelihood of encountering intricate postoperative complications was observed among non-survivors.

**Table 1.** Demographic and clinical variables of the 1049 patients who underwent ATAAD surgery.

Variable	Total (*n* = 1049)	Survivors (*n* = 925)	Non-survivors (*n* = 124)	*P* value
Age (years)	53.02 ± 13.21	52.42 ± 13.02	57.43 ± 13.78	<.001
Gender male *n* (%)	785 (74.83)	695 (75.14)	90 (72.58)	0.538
BMI (kg/m^2^)	25.58 (23.03-28.02)	25.65 (23.03-28.08)	25.43 (23.24-27.77)	0.582
Medical History *n* (%)				
Hypertension	765 (72.93)	673 (72.76)	92 (74.19)	0.735
Diabetes mellitus	38 (3.62)	35 (3.78)	3 (2.42)	0.612
Smoking	243 (23.16)	212 (22.92)	31 (25.00)	0.606
Alcohol drinking	161 (15.35)	141 (15.24)	20 (16.13)	0.797
Preoperative conditions *n* (%)				
Cardiac tamponade	120 (11.44)	93 (10.05)	27 (21.77)	<.001
Cerebral ischaemia	100 (9.53)	83 (8.97)	17 (13.71)	0.092
Limb ischaemia	150 (14.3)	125 (13.51)	25 (20.16)	0.047
Bowl ischaemia	43 (4.1)	38 (4.11)	5 (4.03)	0.968
Myocardial ischaemia	203 (19.35)	163 (17.62)	40 (32.26)	<.001
Operative details *n* (%)				
cannulation *n* (%)				0.328
Ascending aorta/arch cannulation	15 (1.43)	13 (1.41)	2 (1.61)	
Femoral cannulation	222 (21.16)	192 (20.76)	30 (24.19)	
Axillary cannulation	225 (21.45)	206 (22.27)	19 (15.32)	
Femoral + axillary cannulation	577 (55)	504 (54.49)	73 (58.87)	
Root surgery (*n*, %)				
Bentall	218 (20.78)	191 (20.65)	27 (21.77)	0.772
Root reconstruction	788 (75.12)	695 (75.14)	93 (75.00)	0.974
Valve sparing	18 (1.72)	17 (1.84)	1 (0.81)	0.644
Ascending aorta replacement	830 (79.12)	728 (78.70)	102 (82.26)	0.360
Arch surgery *n* (%)				
Hemi arch replacement	202 (19.26)	174 (18.81)	28 (22.58)	0.317
Total arch replacement with FET	493 (47)	432 (46.70)	61 (49.19)	0.602
Arch stent	341 (32.51)	306 (33.08)	35 (28.23)	0.278
Untreated	13 (1.24)	13 (1.41)	0 (0.00)	0.370
Concomitant CABG	68 (6.48)	47 (5.08)	21 (16.94)	<.001
MVP/MVR	15 (1.43)	11 (1.19)	4 (3.23)	0.164
CPB (min)	226.00 (189.00-265.00)	222.50 (187.75-264.00)	241.00 (208.00-293.00)	<.001
CPB ≥ 208 min	640 (61.07)	547 (59.20)	93 (75.00)	<.001
Cross-clamp time (min)	157.00 (126.00-194.25)	156.00 (125.00-193.00)	163.00 (137.75-216.50)	0.300
Cross-clamp time ≥ 216 min	158 (15.08)	127 (13.74)	31 (25.00)	0.001
DHCA time (min)	30.00 (22.00-37.00)	30.00 (22.00-37.00)	32.00 (24.00-36.00)	0.300
DHCA time ≥ 27 min	625 (61.7)	543 (60.87)	82 (67.77)	0.143
Intraoperative transfusion				
Packed red cells (U)	6.00 (4.00-9.00)	5.00 (3.50-9.00)	9.00 (5.88-12.00)	<.001
Packed red cells ≥ 5.25 U	506 (51.95)	419 (48.61)	87 (77.68)	<.001
Fresh frozen plasma (ml)	825.00 (600.00-1000.00)	800.00 (600.00-1000.00)	1000.00 (818.75-1406.25)	<.001
Fresh frozen plasma ≥ 937 ml	391 (40.18)	317 (36.82)	74 (66.07)	<.001
Cryoprecipitate (U)	10.00 (8.00-15.00)	10.00 (8.00-14.00)	12.62 (10.00-18.00)	<.001
Cryoprecipitate ≥ 10.38 U	472 (48.51)	394 (45.76)	78 (69.64)	<.001
Platelets (U)	2.00 (1.00-2.00)	1.00 (1.00-2.00)	2.00 (1.00-2.00)	<.001
Platelets ≥ 1.8 U	498 (51.18)	417 (48.43)	81 (72.32)	<.001
Postoperative data *n* (%)				
Mechanical ventilation time (MVT)	31.00 (14.00-82.33)	28.63 (13.50-70.00)	73.86 (26.80-173.94)	<.001
Mechanical ventilation time (MVT)				<.001
<12 h	194 (18.49)	185 (20.00)	9 (7.26)	
12 h to <24 h	283 (26.98)	262 (28.32)	21 (16.94)	
24 h to <48 h	170 (16.21)	154 (16.65)	16 (12.90)	
48 h to <72 h	119 (11.34)	103 (11.14)	16 (12.90)	
≥72 h	283 (26.98)	221 (23.89)	62 (50.00)	
Tracheostomy	52 (4.96)	34 (3.68)	18 (14.52)	<.001
Haemorrhagic stroke	12 (1.14)	6 (0.65)	6 (4.84)	<.001
Ischaemic stroke	62 (5.91)	45 (4.86)	17 (13.71)	<.001
Paraplegia	21 (2)	17 (1.84)	4 (3.23)	0.487
GI bleeding	17 (1.62)	9 (0.97)	8 (6.45)	<.001
Limb ischaemia	18 (1.72)	11 (1.19)	7 (5.65)	0.001
Bowel ischaemia	17 (1.62)	11 (1.19)	6 (4.84)	0.008
Surgical site infection	36 (3.43)	22 (2.38)	14 (11.29)	<.001
Re-exploration	82 (7.82)	62 (6.70)	20 (16.13)	<.001

Non-survivors, which specifically refers to patients who experienced operative mortality. Abbreviations: BMI: Body mass index; FET: frozen elephant trunk; CABG: Coronary artery bypass graft; MVP/MVR: Mitral valve replacement or Mitral valve repair. CPB: Cardiopulmonary bypass; ACC: Aortic cross-clamp; DHCA: Deep hypothermia circulatory arrest; GI bleeding: Gastrointestinal bleeding.

### Analysis of MVT and operative mortality in ATAAD

**Figure 1.**
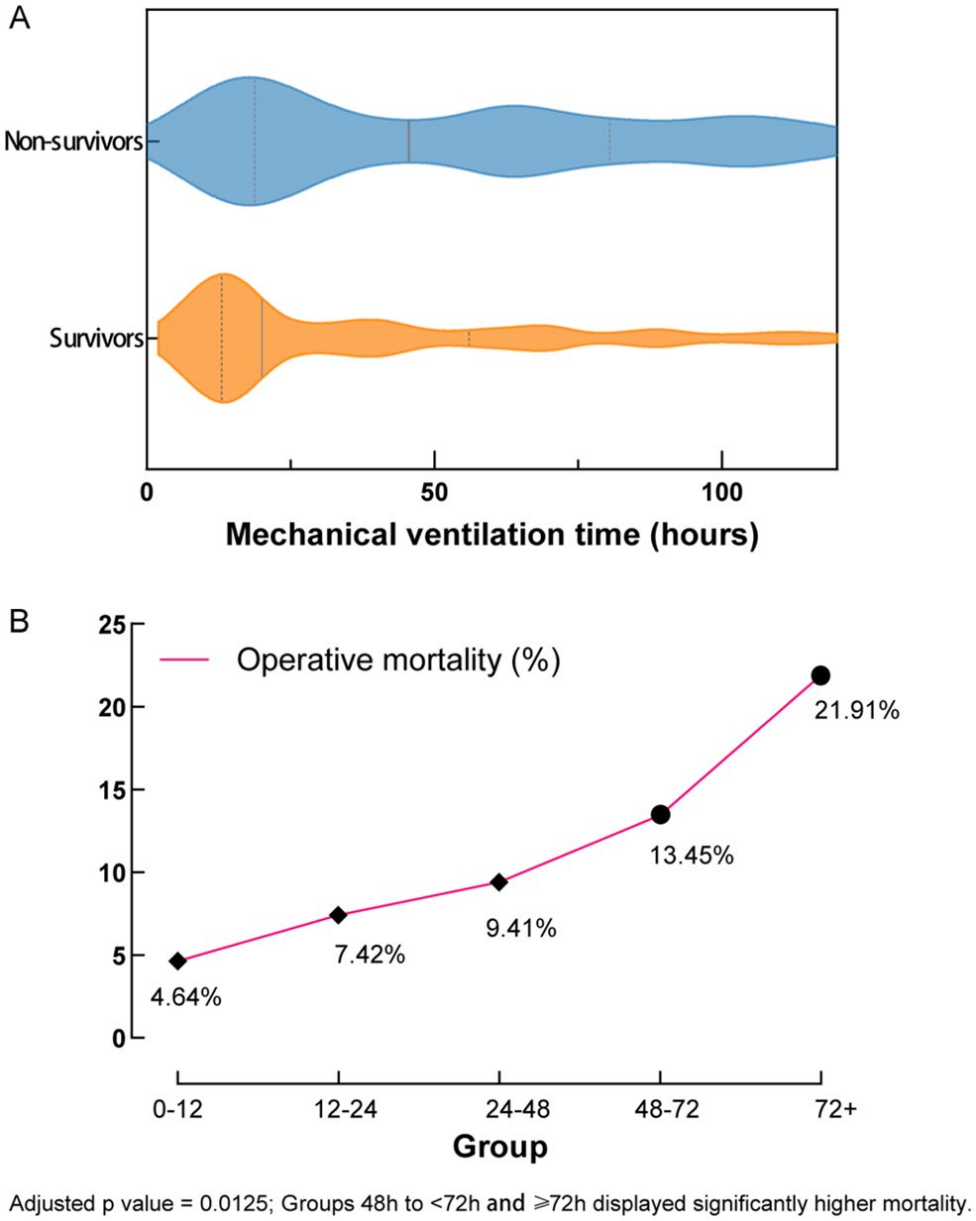
A: the distribution of mechanical ventilation time among survivors versus non-survivors; B: the association between mechanical ventilation time (MVT) subgroups and operative mortality.

The distribution of mechanical ventilation time among survivors versus non-survivors is presented in Figure 1(A) using a violin plot analysis [28.63 (13.50 − 70.00) vs 73.86 (26.80 − 173.94), *p* < 0.001]. Subsequently, subgroups were delineated based on MVT duration: <12 h, 12 h to <24 h, 24 h to <48 h, 48 h to <72 h, and ≥72 h. The association between MVT subgroups and operative mortality was depicted in Figure 1(B). A clear trend emerged, indicating an incremental rise in operative mortality with PMV duration, notably observed in the MVT 48 h to <72 h and MVT ≥ 72 h categories, displaying significantly higher operative mortality in contrast to MVT <12 h.

**Figure 2.**
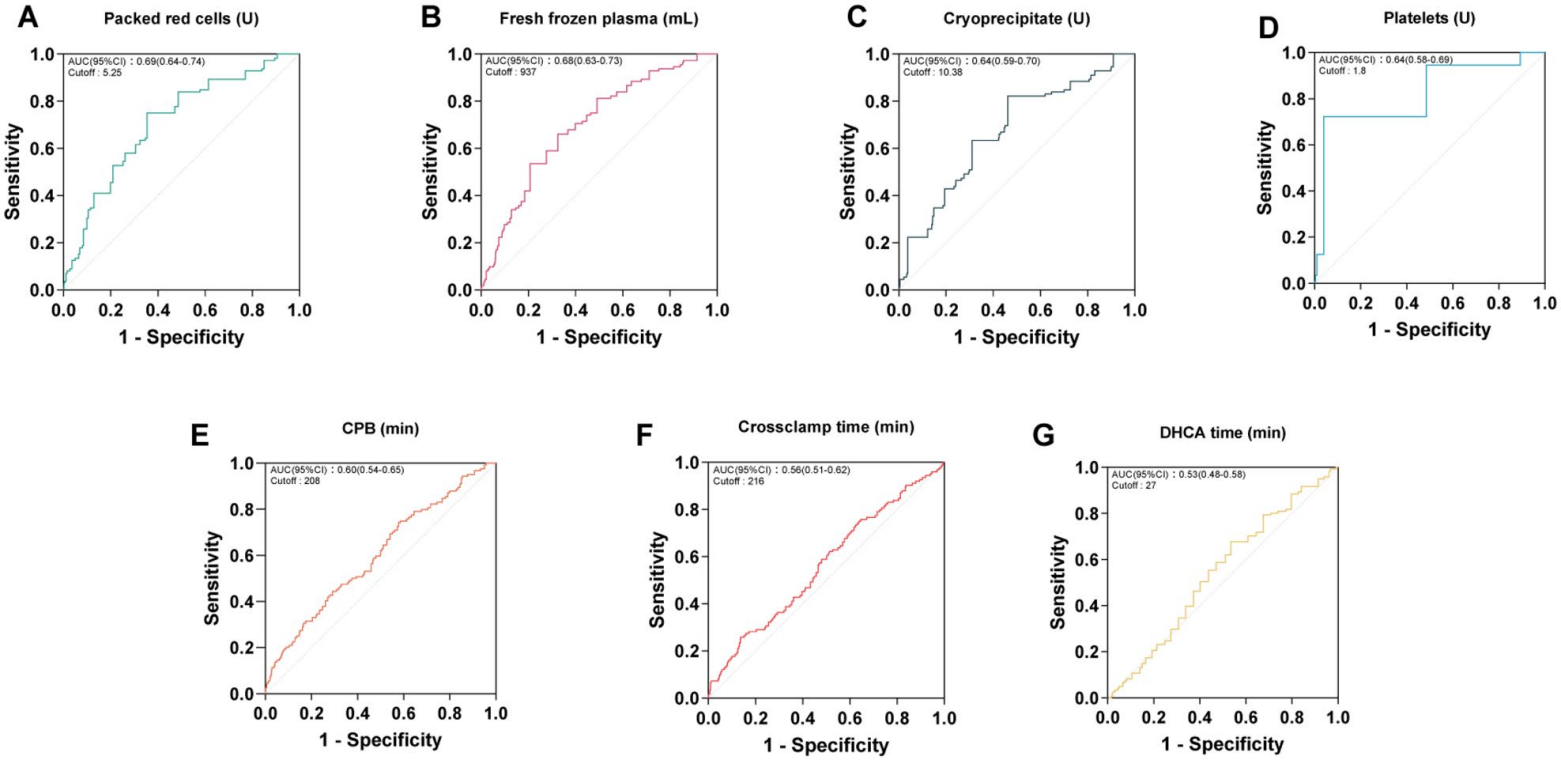
Receiver operating characteristic (ROC) curve analyses depicting the optimal predictive values for surgical duration and intraoperative blood transfusion.

Further investigation employing backward stepwise multivariable logistic regression analysis (refer to Table 2) elucidated the relationship between MVT and operative mortality. Notably, MVT ≥ 72 h exhibited a closely associated high operative mortality (OR: 3.07, 95%CI: 1.54–8.90, *p* = 0.003). This association persisted after adjusting for variables including advanced age (OR: 1.03, 95%CI: 1.01–1.05, *p* < 0.001), cardiac tamponade (OR: 2.72, 95%CI: 1.50–4.91, *p* < 0.001), concomitant CABG (OR: 3.30, 95%CI: 1.60–6.81, *p* = 0.001), prolonged CPB (OR: 1.90, 95%CI: 1.11–3.26, *p* = 0.019), intraoperative transfusions and various postoperative complications. Additionally, no significant difference was observed between MVT 48 h to <72 h and 24 h to <48 h (OR: 1.88, 95%CI: 0.66–5.33, *p* = 0.235), or between MVT ≥ 72 h and 48 h to <72 h (OR: 1.56, 95%CI: 0.78–3.14, *p* = 0.209).

**Table 2.** Backward stepwise multivariable logistic regression for operative mortality after ATAAD repair.

Variable	Odds ratio (95% CI)	*P* value
Ventilation <12 h	ref	ref
12 h to <24 h	2.06 (0.79-5.36)	0.138
24 h to <48 h	2.05 (0.76-5.56)	0.159
48 h to <72 h	2.35 (0.86-6.43)	0.095
≥72 h	3.87 (1.60-9.38)	0.003
Covariates*		
Age (y)	1.03 (1.01-1.05)	<.001
Cardiac tamponade	2.72 (1.50-4.91)	<.001
Concomitant CABG	3.30 (1.60-6.81)	0.001
CPB ≥ 208 min	1.90 (1.11-3.26)	0.019
Packed red cells ≥ 5.25 U	2.05 (1.20-3.50)	0.008
Fresh frozen plasma ≥ 937 ml	2.26 (1.39-3.68)	0.001
Cryoprecipitate ≥ 10.38 U	2.04 (1.24-3.34)	0.005
Haemorrhagic stroke	4.42 (1.07-18.24)	0.040
Ischaemic stroke	2.83 (1.34-5.98)	0.006
GI bleeding	6.40 (1.91-21.45)	0.003
Surgical site infection	3.72 (1.54-9.00)	0.004
Additional comparison*		
48 h to < 72 h vs 24 h to <48 h	1.88 (0.66-5.33)	0.235
≥72 h vs 48 h to <72 h	1.56 (0.78-3.14)	0.209

CI, Confidence interval. *Nonsignificant variables tested: BMI, gender, hypertension, diabetes mellitus, smoking, alcohol drinking, cerebral ischemia, limb ischemia, bowl ischaemia, Myocardial ischaemia, cannulation, Root surgery, Arch surgery, concomitant CABG, concomitant MVP/MVR, cross-clamp time ≥ 216, DHCA time ≥ 27, platelets ≥ 1.8 U, paraplegia, limb ischaemia, re-exploration. Abbreviations: CPB: Cardiopulmonary bypass; GI bleeding: Gastrointestinal bleeding.

**Figure 3.**
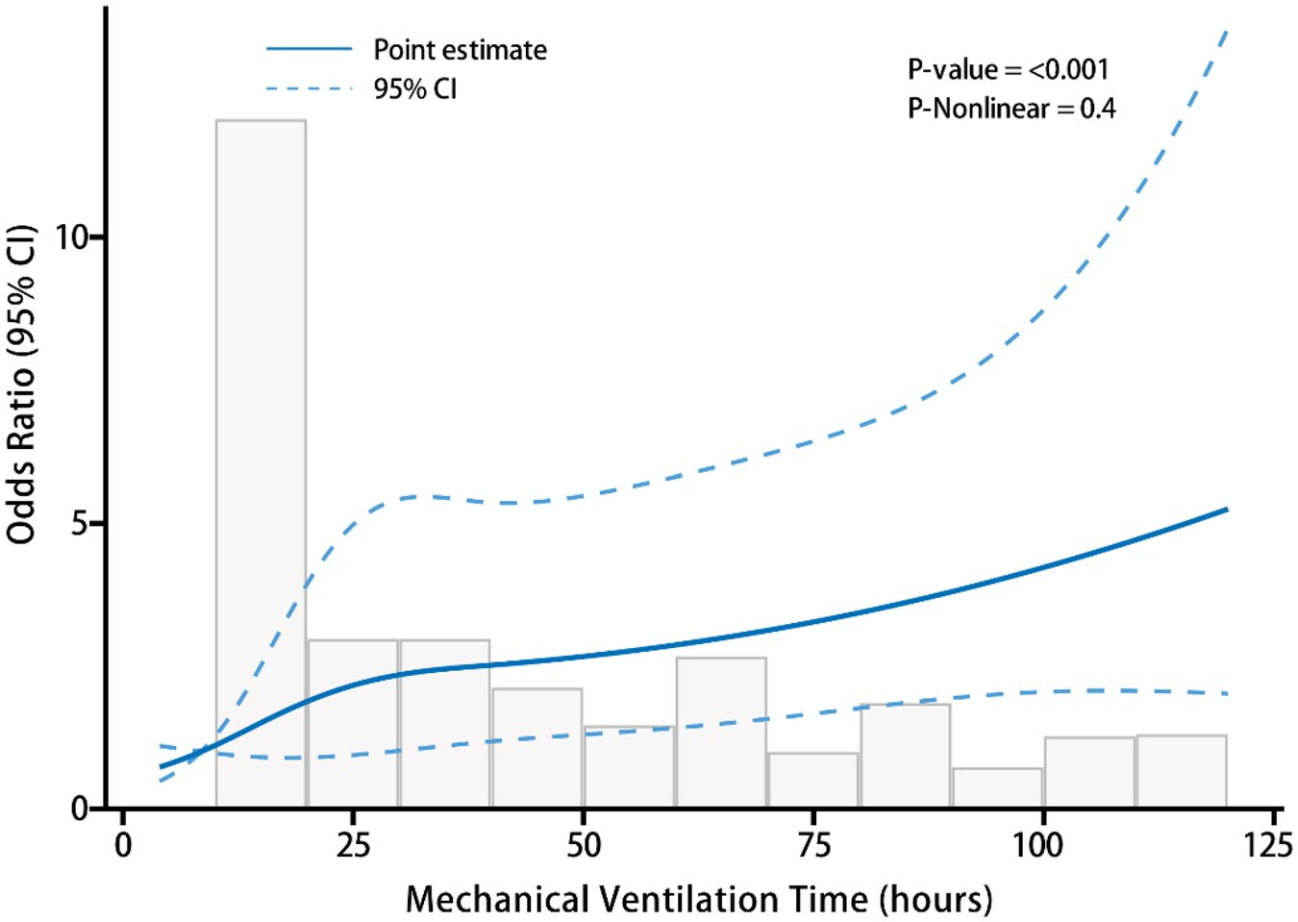
Restricted Cubic spline plot illustrating the relationship between mechanical ventilation duration and operative mortality.

Additionally, we approached MVT as a continuous variable and performed Restricted Cubic Spline (RCS) plot analysis (Figure 3), which illustrated the odds ratio of mortality across MVT ranging from 0 to 125 h. However, RCS analysis indicated no nonlinear association between MVT and operative mortality (P-Nonlinear = 0.4). This comprehensive analysis underscores the significant correlation between PMV, particularly MVT ≥ 72 h, and heightened operative mortality in ATAAD cases.

### Detailed analysis for patients with MVT ≥ 72 h

Among the cohort, 23.89% of survivors (221/925) endured extended mechanical ventilation (MVT ≥ 72 h). Upon comparison (refer to Table 3), it was observed that the MVT ≥ 72 h group exhibited an older age (54.34 ± 13.65 vs 51.81 ± 12.83, *p* = 0.006), with a higher prevalence of preoperative conditions such as cardiac tamponade [41 (14.39%) vs 62 (8.83%), *p* = 0.010] and cerebral ischaemia [37 (12.98%) vs 55 (7.83%), *p* = 0.012]. Prolonged CPB [240.00 (205.00 − 288.00) vs 216.00 (185.00 − 258.00), *p* < 0.001] and cross-clamp times [161.00 (130.00 − 204.00) vs 153.00 (124.00 − 190.00), *p* = 0.005] were noted in group with extended ventilation duration. Additionally, compared to the MVT < 72 h, a higher volume of intraoperative blood transfusion was required in prolonged ventilation group. Postoperatively, patients with MVT ≥ 72 h were more likely to experience complications such as tracheostomy [38 (13.33%) vs 10 (1.42%), *p* < 0.001], stroke [37 (12.98%) vs 22 (3.13%), *p* < 0.001] and re-exploration [33 (11.58%) vs 38 (5.41%), *p* < 0.001].

**Table 3.** Comparison between MVT ≥ 72 h and the counterpart.

Variable	MVT < 72 h (*n* = 702)	MVT ≥ 72 h (*n* = 285)	*P* value
Age (years)	51.81 ± 12.83	54.34 ± 13.65	0.006
Gender male *n* (%)	532 (75.78)	210 (73.68)	0.489
BMI (kg/m^2^)	25.58 (22.99 − 28.20)	25.74 (23.44 − 27.99)	0.631
Medical History *n* (%)			
Hypertension	510 (72.65)	211 (74.04)	0.657
Diabetes mellitus	26 (3.70)	10 (3.51)	0.882
Smoking	170 (24.22)	52 (18.25)	0.042
Alcohol drinking	115 (16.38)	34 (11.93)	0.077
Preoperative conditions n (%)			
Cardiac tamponade	62 (8.83)	41 (14.39)	0.010
Cerebral ischaemia	55 (7.83)	37 (12.98)	0.012
Limb ischaemia	96 (13.68)	38 (13.33)	0.887
Bowl ischaemia	27 (3.85)	13 (4.56)	0.606
Myocardial ischaemia	120 (17.09)	63 (22.11)	0.066
Operative details *n* (%)			
cannulation *n* (%)			<.001
Ascending aorta/arch cannulation	11 (1.58)	5 (1.77)	
Femoral cannulation	152 (21.87)	53 (18.79)	
Axillary cannulation	390 (56.12)	224 (79.43)	
Femoral + axillary cannulation	142 (20.43)	0 (0.00)	
Root surgery (*n*, %)			0.547
Bentall	149 (21.23)	53 (18.60)	
Root reconstruction	10 (1.42)	7 (2.46)	
Valve sparing	526 (74.93)	219 (76.84)	
Ascending aorta replacement	545 (77.64)	235 (82.46)	0.092
Arch surgery *n* (%)			0.955
Hemi arch replacement	130 (18.52)	56 (19.65)	
Total arch replacement with FET	330 (47.01)	135 (47.37)	
Arch stent	233 (33.19)	90 (31.58)	
Untreated	9 (1.28)	4 (1.40)	
Concomitant CABG	31 (4.42)	25 (8.77)	0.007
MVP/MVR	10 (1.42)	3 (1.05)	0.876
CPB (min)	216.00 (185.00 − 258.00)	240.00 (205.00 − 288.00)	<.001
CPB ≥ 208 min	390 (55.63)	208 (72.98)	<.001
Cross-clamp time (min)	153.00 (124.00 − 190.00)	161.00 (130.00 − 204.00)	0.005
Cross-clamp time ≥ 216 min	86 (12.27)	58 (20.35)	0.001
DHCA time (min)	30.00 (22.00 − 38.00)	30.00 (24.00 − 36.00)	0.699
DHCA time ≥ 27 min	415 (60.14)	171 (65.02)	0.167
Intraoperative transfusion			
Packed red cells (U)	5.00 (3.50 − 8.00)	7.50 (4.00 − 10.00)	<.001
Packed red cells ≥ 5.25 U	307 (45.89)	157 (63.31)	<.001
Fresh frozen plasma (ml)	800.00 (575.00 − 975.00)	975.00 (700.00 − 1125.00)	<.001
Fresh frozen plasma ≥ 937 ml	219 (32.78)	137 (55.24)	<.001
Cryoprecipitate (U)	10.00 (8.00 − 14.00)	12.00 (9.44 − 15.00)	<.001
Cryoprecipitate ≥ 10.38 U	300 (44.91)	137 (55.24)	0.005
Platelets (U)	1.00 (1.00 − 2.00)	2.00 (1.00 − 2.00)	<.001
Platelets ≥ 1.8 U	303 (45.36)	152 (61.29)	<.001
Postoperative data *n* (%)			
Mechanical ventilation time (MVT)	17.00 (12.00 − 38.00)	120.00 (96.00 − 182.00)	<.001
Tracheostomy	10 (1.42)	38 (13.33)	<.001
Haemorrhagic stroke	3 (0.43)	5 (1.75)	0.086
Ischaemic stroke	22 (3.13)	37 (12.98)	<.001
Paraplegia	7 (1.00)	12 (4.21)	<.001
GI bleeding	7 (1.00)	5 (1.75)	0.507
Limb ischaemia	6 (0.85)	10 (3.51)	0.007
Bowel ischaemia	6 (0.85)	6 (2.11)	0.192
Surgical site infection	14 (1.99)	18 (6.32)	<.001
Re-exploration	38 (5.41)	33 (11.58)	<.001

Abbreviations: BMI: Body mass index; FET: frozen elephant trunk; CABG: Coronary artery bypass graft; MVP/MVR: Mitral valve replacement or Mitral valve repair. CPB: Cardiopulmonary bypass; ACC: Aortic cross-clamp; DHCA: Deep hypothermia circulatory arrest; GI bleeding: Gastrointestinal bleeding.

### Multivariate logistic regression model for predicting PMV and development of a nomogram model

**Figure 4.**
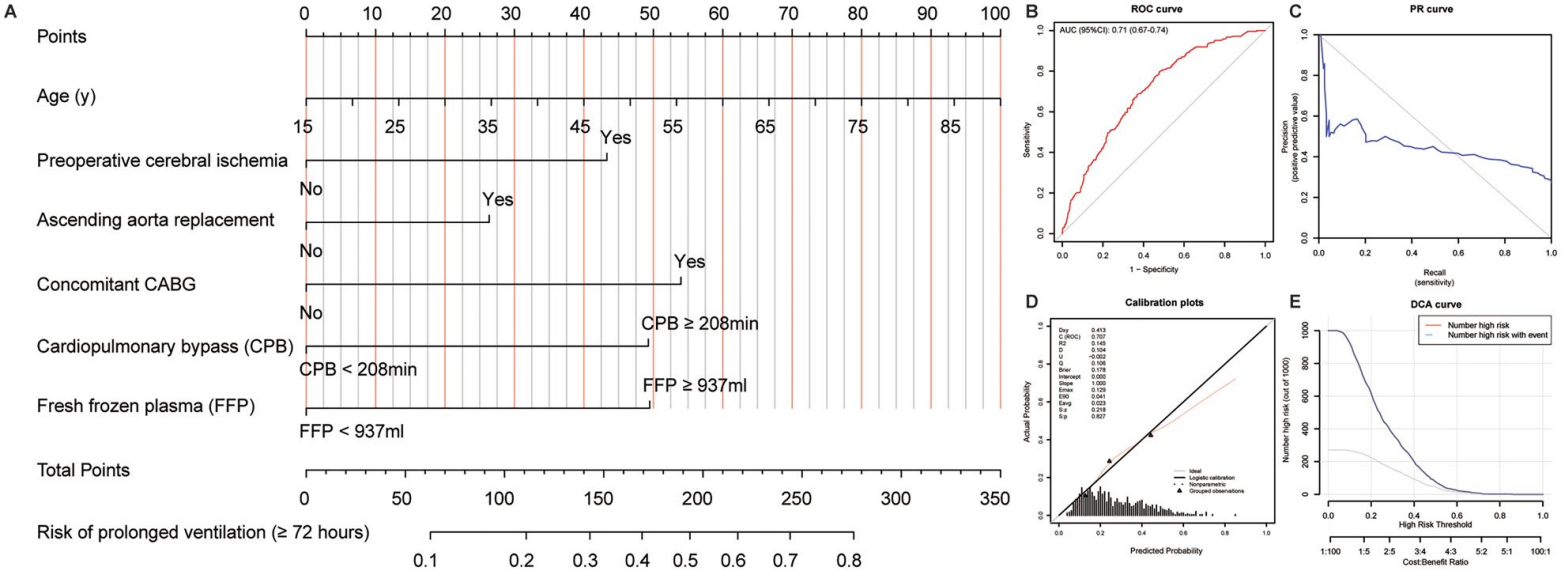
Nomogram model predicting postoperative mechanical ventilation time (MVT) ≥72 h.

A comprehensive backward stepwise multivariable logistic regression analysis revealed strong associations between several variables and MVT ≥ 72 h. Notably, older age (OR: 1.02, 95%CI: 1.01–1.03, *p* = 0.005), preoperative cerebral ischemia (OR: 1.95, 95%CI: 1.19–3.20, *p* = 0.009), ascending aorta replacement (OR: 1.58, 95%CI: 1.03–2.44, *p* = 0.037), concomitant CABG (OR: 2.39, 95%CI: 1.29–4.43, *p* = 0.006), prolonged CPB (OR: 2.31, 95%CI: 1.62–3.30, *p* < 0.001), and higher volume of fresh frozen plasma during surgery (OR: 1.96, 95%CI: 1.40–2.74, *p* < 0.001) were identified as robust predictors strongly associated with MVT ≥ 72 h. Leveraging these findings, a nomogram was devised (refer to Figure 4) wherein each variable’s option was assigned a specific score. The cumulative sum of these scores corresponded to a predictive probability for MVT ≥ 72 h. The model exhibited a C-index of 0.71 (0.67–0.74), signifying a differentiated sensitivity and specificity. Furthermore, calibration plots demonstrated good agreement between the model and actual observations. The DCA curve indicated a high clinical applicability of the nomogram.

### Detailed analysis for patients with MVT < 12 h

A total of 17.79% (185/1040) patients who underwent ATAAD surgery extubated successfully within 12 h. A detailed analysis (refer to Table E1 in the attached document) of this cohort revealed that those who achieved successful extubation within 12 h postoperatively were generally younger (50.56 ± 11.68 vs 53.41 ± 13.42, *p* = 0.004), with a predominantly male composition [152 (82.16%) vs 626 (73.22%), *p* = 0.011]. Furthermore, patients with MVT < 12h exhibited significantly shorter CPB times [206.00 (179.00 − 251.00) vs 230.00 (190.00 − 268.75), *p* < 0.001] and lower intraoperative blood transfusion volumes. Patients underwent early extubation following ATAAD surgery demonstrated a lower tracheostomy rate [1 (0.54%) vs 50 (5.85%), *p* = 0.002] and a reduced incidence of stroke [5 (2.70%) vs 57 (6.67%), *p* = 0.039].

### Multivariable logistic regression model for postoperative tracheostomy

Among the cohort, 4.9% (52/1049) necessitated a tracheotomy, and 18 patient fatalities. Notably, patients experiencing MVT ≥ 72h (OR: 10.59, 95%CI: 1.01–1.06, *p* = 0.006) were significantly associated with requiring a tracheostomy post-ATAAD repair. Additionally, older age (OR: 1.04, 95%CI: 1.01-1.06, *p* = 0.006) and postoperative stroke (OR: 4.45, 95%CI: 2.06–9.61, *p* < 0.001) emerged as covariates associated with tracheostomy (refer to Table 4).

**Table 4.** Backward stepwise multivariable logistic regression for tracheostomy.

Variable	Odds ratio (95% CI)	*P* value
Age (y)	1.04 (1.01–1.06)	0.006
Ventilation <12 h	ref	ref
12 h to <24 h	1.35 (0.24–7.50)	0.732
24 h to <48 h	1.61 (0.26–9.86)	0.605
48 h to <72 h	1.46 (0.20 − 10.62)	0.710
≥72 h	10.59 (2.47–45.41)	0.001
Ischaemic stroke	4.45 (2.06–9.61)	<.001

CI, Confidence interval.

## Discussion

This investigation elucidates several pivotal aspects: 1. Patients undergoing surgery for ATAAD typically endure prolonged periods of mechanical ventilation; 2. Specifically, our focus centred on elucidating the correlation between MVT and operative mortality. Our findings delineate a notable association wherein patients in the MVT ≥ 72 h cohort exhibited a significantly heightened incidence of short-term mortality following ATAAD surgery, while those with MVT < 12 h demonstrated a more favourable prognosis; 3. Through the analysis of clinical data, a nomogram was formulated to forecast PMV subsequent to ATAAD surgery. This predictive model exhibited exceptional accuracy and reliability; 4. Our exploration delved into the relationship between MVT and postoperative tracheotomy. Importantly, our observations underscore a significant correlation between MVT ≥ 72 h and an increased likelihood of necessitating subsequent tracheotomy.

ATAAD constitutes an urgent and potentially fatal condition necessitating immediate surgical intervention [2]. Historically, the mortality rates associated with ATAAD surgery have been documented in the range of 11% to 25%. Notably, the operative mortality rate at our centre between 2011 and 2019 stood at 11.85%, aligning closely with prior published reports on this critical surgical intervention [20–22]. Postoperative pulmonary complications, encompassing PMV, pneumonia, reintubation, and tracheotomy, represent significant challenges following ATAAD repair. These complications, along with other adverse outcomes, were likely to result in devastating incidents [23,24].

Postoperative employment of mechanical ventilation is frequently undertaken to optimize blood oxygen levels and alleviate the myocardial and respiratory muscle burden, commonly applied in patients undergoing ATAAD repair [9]. Moreover, the duration of mechanical ventilation may significantly reflect a patient’s recovery trajectory and the incidence of postoperative complications [3]. For instance, in cases of postoperative cerebral infarction or re-exploration surgery, patients often require mechanical ventilation support to navigate through this critical phase. However, prolonged reliance on mechanical ventilation subsequent to ATAAD surgery can precipitate diverse complications. These complications encompass cardiopulmonary damage, heightened vulnerability to lung infections, prolonged stays in the intensive care unit, and escalated healthcare expenditures [10]. Consequently, exploring the role of mechanical ventilation duration following ATAAD repair assumes paramount importance in comprehending its implications and optimizing patient outcomes.

In conventional cardiac surgery, numerous studies have shown that PMV is a risk factor for adverse outcomes such as postoperative mortality. PMV is typically defined as extubation occurring 24 or 48 h after surgery [14,25,26]. However, ATAAD has distinct pathological, physiological, and treatment characteristics compared to conventional heart surgery. The emergence of acute pain, coupled with CPB and DHCA during surgery, amplifies systemic inflammatory responses, elevating the risk of postoperative pulmonary complications [4,11,12]. Consequently, a notable incidence of postoperative hypoxemia was observed, ranging between 30% to 67.6% among patients undergoing ATAAD repair. Remarkably, a significant proportion continued to experience hypoxia even 72-h post-surgery [27,28]. This circumstance prompted us to question the suitability of the conventional 48-hour delayed extubation benchmark for patients following ATAAD surgery. Hence, our study sought to investigate the impact of postoperative intubation duration on outcomes in ATAAD and delineate an appropriate threshold for prolonged postoperative ventilation time. In this pursuit, we refined the time intervals for auxiliary ventilation. These intervals were delineated as follows: <12 h, 12 to <24 h, 24 to <48 h, 48 to <72 h, and ≥72 h. This categorization aimed to intricately elucidate the relationship between different ventilation timeframes and the corresponding prognosis. Our findings unveiled markedly favourable outcomes associated with ventilation durations of less than 12 h. Conversely, a conspicuous correlation emerged between MVT ≥ 72 h and heightened mortality rates in ATAAD surgery. Notably, while mortality rates exhibited variability within the 12 to 72-hour range, the overarching correlation with prognosis remained consistent.

In contrast to preceding studies, we refined the categorization of postoperative ventilation time by introducing two additional groups: a 48 to 72-hour range and a group exceeding 72 h. While existing literature indicates a stronger association between postoperative assisted ventilation surpassing 48 h and escalated mortality, our investigation revealed a more pronounced correlation with durations ≥72 h. Considering a cutoff value of mechanical ventilation surpassing 72 h as indicative of ‘delayed extubation’ subsequent to ATAAD surgery, prior research has documented incidence rates ranging from 10.2% to 25% [3,5,29]. Notably, our observed incidence rate stands notably higher at 26.98%. This disparity might be attributed to the heightened complexity of cases in our study cohort, with 19.35% presenting preoperative myocardial ischemia and 47% undergoing total arch replacement with insertion of a prosthetic graft (frozen elephant trunk, FET).

Our research findings, in conjunction with existing literature, bring into question the applicability of postoperative rapid recovery strategies, specifically ERPs, for patients undergoing surgery for ATAAD. Traditional rapid recovery protocols advocate for extubation within 6 h post-cardiac surgery [30]. However, the distinctive pathological and physiological features inherent in ATAAD highlight a heightened occurrence of postoperative respiratory system-related complications, posing inherent risks to immediate extubation [31]. Importantly, our study does not negate the concept of ERPs. Instead, it furnishes more compelling evidence supporting postoperative recovery strategies tailored to ATAAD surgical patients. Our research indicates that achieving stable outcomes involves safely extubating patients within 72 h following ATAAD surgery. This emphasizes the imperative need for a spectrum of postoperative respiratory rehabilitation strategies. Incorporating timely interventions such as inhaled nitric oxide and sequential non-invasive ventilation becomes crucial to enable more patients to enter the initial 72-hour extubation window post-surgery [19,28]. This approach allows for a more flexible duration of postoperative ventilation, aligning with the pathophysiological alterations in the lungs post-ATAAD surgery, particularly in resolving pulmonary oedema and mitigating inflammation [8,28,32].

An essential consideration lies in predicting the likelihood of delayed extubation subsequent to ATAAD surgery. Both preoperative and intraoperative factors significantly contribute to the occurrence of postoperative delayed extubation [33]. Our study identified several critical factors associated with this delay. Factors such as age, pericardial tamponade, myocardial ischemia, concurrent CABG, prolonged bypass time or clamp time, and substantial blood transfusion emerged as high-risk indicators for mortality. Likewise, these same factors—age, pericardial tamponade, cerebral ischemia, concurrent CABG, prolonged bypass time or clamp time, and significant blood transfusion—also constitute risk factors for PMV.

Advanced age consistently stands as an acknowledged risk factor associated with both short-term and long-term outcomes [34]. Despite adjustments made for confounding factors, several studies underscore the significant association between preoperative tamponade and heightened postoperative mortality [2,35]. Moreover, prolonged CPB duration and extensive intraoperative blood transfusion requirements underscore the intricate nature of the surgery, unequivocally linked to postoperative mortality [6,16]. Concurrently, prolonged surgical procedures amplify the susceptibility to postoperative infections, closely tied to unfavourable prognosis [36]. While prior research predominantly emphasized postoperative ischemic stroke following ATAAD surgery, our study diverges by highlighting the elevated risk of mortality associated with postoperative haemorrhagic stroke compared to ischaemic stroke. This discernment may be attributed to the complexity of aortic arch procedures, prolonged CPB duration, and extended periods of aortic clamping [37].

Based on backward stepwise regression analysis incorporating multiple factors, we established a model comprising six variables in the form of a nomogram for convenient practical application. This model integrates factors such as advanced age, preoperative cerebral ischaemia, ascending aorta replacement, concurrent CABG, prolonged CPB duration, and higher volume of intraoperative FFP requirement. Studies indicate that advanced age is a notable risk factor for postoperative hypoxemia [38]. The presence of preoperative cerebral ischemia hints at potential arch involvement, necessitating more intricate repair approaches [2]. Simultaneously, concurrent CABG and the escalation in blood transfusion volumes indicate a more intricate intraoperative course. This frequently leads to extended CPB durations, potentially triggering the activation of leukocytes, complement, platelets, and the coagulation system. Excessive activation of inflammatory cascade may result in heightened capillary membrane permeability, reduced perfusion, and tissue hypoxia, consequently precipitating multi-organ dysfunction, including respiratory impairment, and ultimately contributing to PMV [9,29,39,40]. Furthermore, blood transfusion itself carries an inherent risk of inducing transfusion-associated lung injury, which can subsequently lead to impaired oxygenation [29]. It’s noteworthy that replacement of the ascending aorta emerged as another factor associated with PMV subsequent to ATAAD surgery. This observation possibly stems from our center’s conservative surgical approach tailored for critically ill patients, aiming to achieve favorable short-term outcomes [16]. In summary, the aforementioned risk factors collectively contribute to the occurrence of PMV. Our predictive model has exhibited excellent performance in estimating the probability of postoperative PMV [C-index: 0.71 (0.67–0.74)], providing a valuable tool for clinical decision-making and patient care management.

## Conclusion

In conclusion, PMV is a common complication following ATAAD surgery, with mortality rates significantly increasing when MVT exceeds 72 h. Our study developed a robust nomogram based on a large patient cohort to predict PMV after ATAAD surgery. This predictive model enables healthcare providers to identify high-risk patients, optimize postoperative care, and implement targeted interventions to reduce MVT within 72 h, ultimately improving patient outcomes and prognosis.

## Limitations

The retrospective nature of our study potentially introduces a degree of bias into our conclusions. However, being a high-capacity center, the evidence we present carries substantial reliability. Our investigation primarily delves into the correlation between postoperative mechanical ventilation duration and short-term outcomes in ATAAD cases. Nevertheless, the lack of detailed documentation regarding postoperative pulmonary complications hinders a comprehensive interpretation of our findings. Notably, we selected surgical mortality as the primary endpoint, offering a reasonably accurate portrayal of overall postoperative status. Moreover, the retrospective nature of the study led to missing data due to incomplete medical records. However, given the large sample size and rigorous analysis, we concluded that the missing values did not significantly affect our findings, and thus proceeded without imputation or adjustment [41]. It’s crucial to note that our study’s sample population comprises individuals of Chinese ethnicity. Thus, exercising caution in extrapolating our research conclusions to other populations is essential, necessitating further large-scale studies for broader generalization.

## Supplementary Material

Supplemental Material

## Data Availability

The published article contains all the data that was generated and analysed. If anyone requests the data, they can contact the corresponding author whose contact information is provided on the title page of the article.
