# Supplementary material for: A novel nomogram for predicting prolonged mechanical ventilation after acute type A aortic dissection surgery: a retrospective study investigating the impact of ventilation duration on postoperative outcomes
Source: Ann Med. 2024 Aug 22;56(1):2392871. doi: 10.1080/07853890.2024.2392871 (PMC11342815; doi:10.1080/07853890.2024.2392871)
Supplement: Supplemental Material [file IANN_A_2392871_SM9856.docx]

Table E1 Comparison between MVT ≥ 12 hours and the counterpart

| Variable | MVT < 12 hours  (n = 185) | MVT ≥ 12 hours  (n = 855) | P value |
| --- | --- | --- | --- |
| Age (years) | 50.56 ± 11.68 | 53.41 ± 13.42 | 0.004 |
| Gender male n (%） | 152 (82.16) | 626 (73.22) | 0.011 |
| BMI (kg/m2) | 25.35 (22.63 - 27.68) | 25.71 (23.14 - 28.09) | 0.068 |
| Medical History n (%） |  |  |  |
| Hypertension | 134 (72.43) | 623 (72.87) | 0.904 |
| Diabetes mellitus | 5 (2.70) | 33 (3.86) | 0.447 |
| Smoking | 51 (27.57) | 190 (22.22) | 0.118 |
| Alcohol drinking | 37 (20.00) | 122 (14.27) | 0.050 |
| Preoperative conditions n (%） |  |  |  |
| Cardiac tamponade | 16 (8.65) | 100 (11.70) | 0.233 |
| Cerebral ischemia | 8 (4.32) | 91 (10.64) | 0.008 |
| Limb ischemia | 22 (11.89) | 125 (14.62) | 0.334 |
| Bowl ischemia | 4 (2.16) | 39 (4.56) | 0.137 |
| Myocardial ischemia | 36 (19.46) | 164 (19.18) | 0.931 |
| Operative details n (%） |  |  |  |
| cannulation n (%） |  |  | <.001 |
| Ascending aorta/arch cannulation | 5 (2.70) | 11 (1.30) |  |
| Femoral cannulation | 48 (25.95) | 170 (20.12) |  |
| Axillary cannulation | 28 (15.14) | 624 (73.85) |  |
| Femoral + axillary cannulation | 104 (56.22) | 40 (4.73) |  |
| Root surgery (n, %) |  |  | 0.584 |
| Bentall | 40 (21.62) | 176 (20.58) |  |
| Root reconstruction | 40 (21.62) | 644 (75.32) |  |
| Valve sparing | 5 (2.70) | 13 (1.52) |  |
| Ascending aorta replacement | 124 (67.03) | 700 (81.87) | <.001 |
| Arch surgery n (%） |  |  | 0.336 |
| Hemi arch replacement | 42 (22.70) | 157 (18.36) |  |
| Total arch replacement with FET | 79 (42.70) | 409 (47.84) |  |
| Arch stent | 63 (34.05) | 277 (32.40) |  |
| Untreated | 1 (0.54) | 12 (1.40) |  |
| Concomitant CABG | 7 (3.78) | 58 (6.78) | 0.126 |
| MVP/MVR | 1 (0.54) | 14 (1.64) | 0.427 |
| CPB (min) | 206.00 (179.00 - 251.00) | 230.00 (190.00 - 268.75) | <.001 |
| CPB ≥ 208min | 89 (48.11) | 546 (63.93) | <.001 |
| Crossclamp time (min) | 151.00 (125.00 - 180.00) | 158.00 (126.00 - 196.00) | 0.074 |
| Crossclamp time ≥ 216min | 21 (11.35) | 133 (15.57) | 0.143 |
| DHCA time (min) | 29.50 (22.00 - 38.00) | 30.00 (23.00 - 37.00) | 0.877 |
| DHCA time ≥ 27min | 105 (58.33) | 516 (62.47) | 0.301 |
| Intraoperative transfusion |  |  |  |
| Packed red cells (U) | 5.00 (3.12 - 6.38) | 6.00 (4.00 - 9.50) | <.001 |
| Packed red cells ≥ 5.25 U | 81 (44.51) | 417 (53.26) | 0.033 |
| Fresh frozen plasma (ml) | 750.00 (550.00 - 950.00) | 875.00 (625.00 - 1000.00) | <.001 |
| Fresh frozen plasma ≥ 937ml | 49 (27.07) | 334 (42.66) | <.001 |
| Cryoprecipitate (U) | 10.00 (8.00 - 14.00) | 10.25 (8.25 - 15.00) | 0.129 |
| Cryoprecipitate ≥ 10.38U | 78 (43.09) | 389 (49.68) | 0.110 |
| Platelets (U) | 1.00 (1.00 - 2.00) | 2.00 (1.00 - 2.00) | 0.008 |
| Platelets ≥ 1.8U | 76 (41.99) | 414 (52.87) | 0.008 |
| Postoperative data n (%） |  |  |  |
| Mechanical ventilation time (MVT) | 9.00 (6.50 - 11.00) | 44.00 (18.75 - 95.85) | <.001 |
| Tracheostomy | 1 (0.54) | 50 (5.85) | 0.002 |
| Hemorrhagic stroke | 1 (0.54) | 11 (1.29) | 0.630 |
| Ischemic stroke | 5 (2.70) | 57 (6.67) | 0.039 |
| Paraplegia | 0 (0.00) | 20 (2.34) | 0.071 |
| GI bleeding | 1 (0.54) | 16 (1.87) | 0.330 |
| Limb ischemia | 2 (1.08) | 16 (1.87) | 0.663 |
| Bowel ischemia | 3 (1.62) | 14 (1.64) | 1.000 |
| Surgical site infection | 3 (1.62) | 32 (3.74) | 0.147 |
| Re-exploration | 10 (5.41) | 72 (8.42) | 0.168 |

Abbreviations: BMI, Body mass index; FET, frozen elephant trunk; CABG, Coronary artery bypass graft; MVP/MVR, Mitral valve replacement or Mitral valve repair. CPB, Cardiopulmonary bypass; ACC, Aortic cross-clamp; DHCA, Deep hypothermia circulatory arrest; GI bleeding, Gastrointestinal bleeding.
